# Tailored videoconferencing counselling program to support family carers of people living with dementia during the transition to permanent residential care: a pilot and feasibility randomised trial

**DOI:** 10.1186/s12877-024-04907-4

**Published:** 2024-04-26

**Authors:** Deborah Brooks, Katy Wyles, Nancy A. Pachana, Elizabeth Beattie, Joseph E. Gaugler

**Affiliations:** 1 School of Nursing, Queensland University of Technology, Brisbane, Australia; 2 The University of Queensland Centre for Clinical Research, Brisbane, Australia; 3 School of Psychology, The University of Queensland, Brisbane, Australia; 4 School of Public Health, University of Minnesota, Minneapolis, MN USA

**Keywords:** Dementia, Residential care, Nursing home, Transition, Counselling, Psychosocial support, Family carers, Videoconferencing

## Abstract

**Background:**

Decisions surrounding the permanent residential care placement of people living with dementia can be stressful and distressing; however, providing access to targeted information and support prior to placement may help carers better cope. This mixed methods study aimed to test the feasibility, acceptability, and potential benefits of providing a tailored, individual counselling program (the Residential Care Transition Module), delivered via videoconferencing, to Australian family carers of a relative with dementia during the transition to permanent residential care.

**Methods:**

A total of 18 family carers were randomly allocated to receive either the counselling intervention (six sessions delivered over 12 weeks) or a check-in call, delivered by a trained Transition Counsellor. Both groups received help-sheets about residential care, coping with placement, and managing feelings. Carers completed online surveys about stress, guilt, anxiety, depression, grief, and support for caring at baseline and four months post-baseline. Carers were also invited to participate in semi-structured exit interviews, conducted after follow-up surveys were completed. Process data relating to recruitment, retention, intervention dose and delivery were collected via logs. Quantitative data were analysed using descriptive statistics and repeated measures ANOVA. Qualitative data, relating to feasibility, acceptability, and perceived benefits of the program, were analysed using the ‘framework’ approach developed by the Medical Research Council to inform the process evaluation of complex interventions.

**Results:**

Qualitative findings indicated that delivery of the counselling program during the transition period was deemed by participants to be feasible and acceptable. Delivery via videoconferencing was deemed convenient and acceptable, with few technical issues. The skills and knowledge of the Transition Counsellor were perceived to be important mechanisms of impact. Though not statistically significant, promising quantitative findings were identified in terms of reduced carer stress and guilt and improved support for caring.

**Conclusions:**

Delivery of a tailored counselling program via videoconferencing to family carers of people living with dementia during the transition to residential care was feasible and acceptable. The program has the potential to improve transitional support to family carers.

**Trial registration:**

This study was registered in the Australian New Zealand Clinical Trials Registry: ACTRN12621001462875.

## Background

Whilst most people living with dementia reside in the community, up to two-thirds may eventually require residential or nursing home care until end of life [1, 2]. The transition from living at home to residential care involves several ‘processes’ for family carers to navigate over a period of time, including decision-making to place into care, finding a suitable care facility, assessment and admission processes, moving the person with dementia into the facility, and settling in [3]. Factors influencing outcomes during this transitional period include the degree of pressure, choice, control, information, preparedness, and practical and emotional support that carers have [4, 5]. Stressors may include difficulties finding a suitable residential care community, lack of financial assistance, dealing with complex administrative processes, and lack of support from family and/or health and social care professionals either in relation to the decision to place or the admission/moving in process itself [4, 6]. Unsurprisingly, many families describe this time as being incredibly difficult, highlighting the need for improved psychosocial support during the transition period [3, 6]. Research into transitional psychosocial supports for family carers has been limited to date however [7], with most research focusing on interventions to delay or prevent admission to long-term care homes [8]. The rationale for this research was therefore to ‘bridge the support void’ that family carers often experience when their relative with dementia transitions into permanent residential aged care, specifically during the time from assessment and approval to admission into a residential care home.

### Need for transitional support for carers

Research internationally has highlighted the need for better emotional and practical support to be provided to families caring for a person living with dementia, and especially during care transitions [9, 10]. The need for such psychosocial support does not end when making decisions about residential care or nursing home placement [11–13]. In Australia, older adults typically receive a formal assessment of need by an Aged Care Assessment Team (ACAT), comprising qualified staff such as doctors, nurses, social workers, psychologists, and allied health professionals, to determine eligibility for residential care. Once approval has been received however, most families are left to navigate the decision-making and admission processes themselves [3]. Residential care placement itself may be planned or unplanned, with crisis admissions for example following the hospitalisation of the person living with dementia, being particularly stressful [5]. Regardless of the context, families have repeatedly reported feelings of stress, anxiety, depression, guilt, and grief during the transition period [5, 14–16], with little formal support for those carers who struggle to cope [3, 6, 17]. Lack of formal support pre-placement may partly explain the moderate to high levels of depression, grief, and stress reported by some family carers post-placement [18]. It may also help explain why residential care staff can feel that some family carers are poorly prepared for placement, lack knowledge about the natural progression of dementia and have unrealistic expectations about the provision of care within the facility [11].

### Options for the provision and delivery of transitional support

The types of support identified as needed during the placement transition period include the provision of appropriate and timely information about residential care provision and admission processes, psychoeducation about the progression of dementia, emotional and social support, skills in communication, assertiveness, and self-care [3, 11]. Depending on local services, transitional supports to family carers could be provided by a range of health and social care professionals, such as social workers, psychologists, counsellors, community psychiatric nurses, dementia key workers, aged care system navigators, and dementia carer organisations [11]. Access to and use of such support services however, often depends on the geographical location of the service in relation to the carer (i.e., whether living in urban, regional, or remote areas), the carer’s ability to leave their family member to attend face-to-face sessions, and/or their access to (as well as adoption of and support to use) the internet and related technologies for services provided by telehealth (e.g., suitable device, videoconferencing software) [19, 20]. Notably, telehealth (telephone and/or videoconferencing) delivery of psychosocial interventions for both people living with dementia and their families necessarily increased during and following the COVID19 pandemic with promising results [21].

Whilst a number of research studies have trialled psychosocial interventions delivered via telehealth to address the need for carer support in the months immediately post-residential care placement [18, 22–24], there are currently no specific psychosocial support programs available to assist Australian families caring for someone living with dementia earlier in the transition process to residential care. The Residential Care Transition Module (RCTM) is an evidence-based multicomponent psychosocial intervention originally developed in the United States (US) to be delivered to family carers of people living with dementia in the post-placement period [22, 23]. Following successful cultural adaptation of the RCTM to an Australian context [18], we hypothesised that delivery of the program in the transitional time period may ease the process itself and help family carers better cope and adjust once their relative has been admitted into residential care. Australia differs from the US in that formal assessment of need and approval from an ACAT is necessary for Government subsidised residential care placement; two thirds of Australians living with dementia are placed into residential care within 24 months of this assessment [1]. Australian data indicates that in 2018–2019 there were 200,700 ACAT approvals for permanent residential care and 60,000 admissions, with the median wait-time in-between ACAT approval and residential care placement being 152 days [25]. Additionally, 40% of people approved for permanent residential care had a wait-time of nine months or more [25]. This presents an opportunity to provide psychosocial support to carers of people living with dementia approved, and potentially on the waitlist, for residential care placement, to help cope with the placement process itself. Given the geographical landscape of Australia, videoconferencing delivery of counselling support also presents opportunities to increase access. The aim of this study was therefore to test the feasibility and acceptability of delivering a tailored videoconferencing counselling program specifically designed to ease the residential care transition process for families of people living with dementia.

### Aims and research questions

We tested whether it was feasible and acceptable to conduct a randomised controlled trial of the RCTM delivered to family carers after ACAT approval for residential care and during the placement process itself. Specifically, this pilot intervention study aimed to test the following: feasibility of participant recruitment and retention, feasibility of intervention delivery and data collection, acceptability of the intervention to carers, and preliminary effects on carer perceived stress, anxiety, guilt, depression, grief, and socio-emotional support.

We aimed to address the following research questions:

1. How appropriate and effective are strategies for recruiting family carers of people living with dementia following ACAT approval and before residential care admission? Are eligible participants willing to be randomised, and potentially be allocated to the non-intervention trial arm?
2. Is delivery of the RCTM intervention following ACAT assessment approval for residential care, feasible and acceptable?
3. What are the rates of retention and attrition?
4. What are the preliminary effects of the RCTM on measures of carer perceived stress, anxiety, guilt, grief, depression, and socio-emotional support?
5. Is the intervention perceived to be helpful from the participant’s perspective?
6. Was the timing of the intervention perceived to be beneficial?

### Conceptual/theoretical models

The theoretical framework underpinning the RCTM is the Expanded Stress Process Model of Family Caregiving in Institutional Settings [26]. This model includes stressors specific to family carers placing a relative into long-term institutional care, such as changes in the carer and resident relationship as a result of placement, carer and resident adjustment to placement, and nursing home stressors such as communication and interaction with staff, other residents and their families, staff support, and interactions with the resident whilst visiting [26]. According to this model, the RCTM intervention aims to boost the internal resources available to the carer (i.e., the mediating processes of cognitive appraisal, problem-focused coping, emotion-focused coping, support-seeking and acceptance) by providing emotional and practical support and with direction to other external resources as necessary.

The Medical Research Council guidance (MRC) for process evaluation of complex intervention was utilised for qualitative analysis purposes. This framework defines complex interventions as having several interacting components (intervention complexity), mediators or moderators (pathway complexity) and/or range of possible outcomes (outcome complexity; [27].

## Methods

### Study design and ethics

The feasibility study was a small-scale parallel randomised controlled trial (RCT) of an individualised evidence-based psychosocial counselling intervention (RCTM) delivered by videoconferencing (e.g., via Zoom) versus a one-off check-in call comparison group. Both groups were emailed a standardised information pack consisting of seven PDF help sheets which are freely available from Dementia Australia. This study was approved by Queensland University of Technology Human Research Ethics Committee (HREC) [2021000194] and registered in the Australian New Zealand Clinical Trials Registry on 26/10/2021: ACTRN12621001462875.

### Participant sample

As this was a pilot feasibility trial and given the known difficulties in recruiting carers of people with dementia into research studies, the target sample size for this study was 30 (15 carers per arm). No formal sample size calculations were undertaken due to the more exploratory nature of the study, however, 10–30 participants per arm is often suggested as being acceptable for feasibility studies [28]. Inclusion criteria for the study were: Any family member (related biologically or by marriage or choice) who was a primary carer of a person living with dementia who had received approval from an ACAT for residential care (but not yet moved into permanent care at study enrolment); English-speaking with sufficient hearing ability to participate in counselling sessions; Over 18 years of age. Family carers needed to be English-speaking, able to read and understand recruitment letters and information sheets or have someone to read these for them to take part in the study. Those in the intervention group needed to be able to hear sufficiently to take part in videoconferencing intervention sessions, which may require the use of a hearing aid or assistive technology device (e.g., hearing assist telephone) to take part. Eligibility was checked upon carers contact with the research team to express interest in the study.

### Recruitment methods

Recruitment of family carers to research studies, especially at times of stress, can be difficult. We planned to use several recruitment strategies including advertisements in social media, newsletters from key carer and community organisations, flyers sent to respite care and day care facilities, and via ACATs. We also used the Australian StepUp for Dementia Research registry as a recruitment tool [29]. This is an online self-registration service that enables volunteers with memory problems or dementia, carers of those with memory problems or dementia, and healthy volunteers to register their interest in taking part in research. Carers replied directly to the research team if interested in taking part, and to discuss eligibility and ask questions as part of the informed consent process. The research team sent out information sheets and consent forms as appropriate. Carers were encouraged to discuss participation with family, friends, or professionals before signing and could withdraw from the study at any time and without giving a reason. Spoken consent was re-established at the beginning of each intervention session and online consent was provided at each data collection point. Recruitment took place between January 2022 and February 2023, within the funded time period.

### Randomisation and blinding

Randomisation was on a 1:1 basis by a statistician blinded to participant identification using a computer-generated allocation sequence which was applied after baseline data was obtained. The research assistant collecting/managing survey data was blinded to group assignment. The Trial Manager assigned participants to groups and so was necessarily un-blinded to participant group assignment. Carers were aware of whether they were receiving the RCTM intervention and so were also not blinded. A trained Transition Counsellor delivered the intervention by videoconferencing to those in the intervention group and provided a check-in call to those in the comparison group but was not involved in collecting baseline or outcome data. There was no replacement of drop-outs due to resource constraints. This data was important in terms of the feasibility of intervention delivery and retention in the study.

### RCTM intervention group

**Fig. 1:**
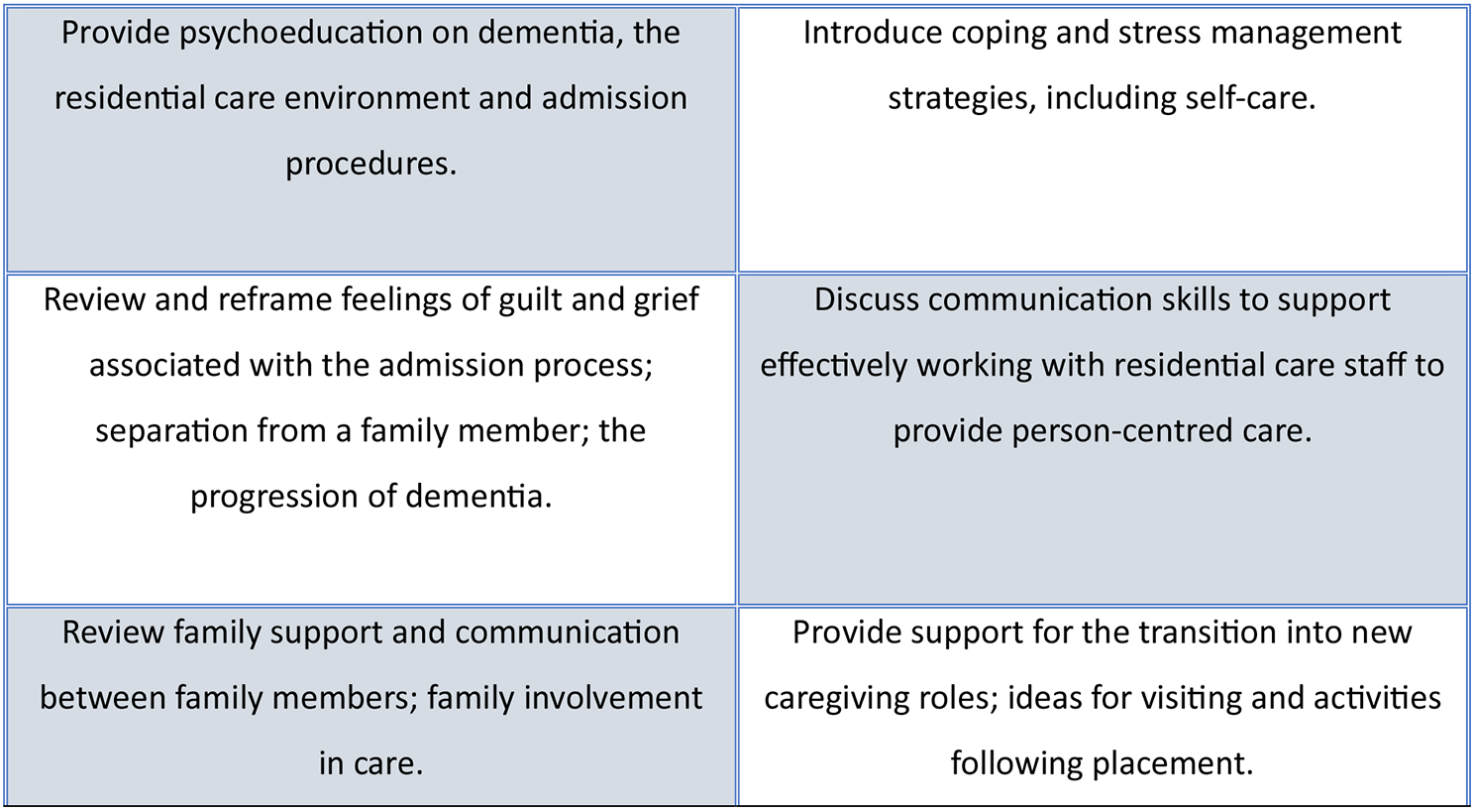
Components of the Residential Care Transition Module

The RCTM is an evidence-based intervention designed to help family carers of people living with dementia manage the potential stressors and issues that arise from admitting a relative into residential care [22, 23]. Those allocated to the intervention group were invited to participate in six individualised videoconferencing counselling sessions, over a period of 12 weeks. These were delivered by a trained Transition Counsellor (registered mental health counsellor with knowledge and experience of dementia and caregiving, and Australian residential aged care facilities, and with educational and experiential grounding in grief counselling and in stress management techniques), using a detailed intervention protocol which included several components as shown in Fig. 1 (adapted from [30].

RCTM components included processing the carer’s experience and helping to review and validate the decision to admit their relative into residential care; assessment of individual needs and key issues; problem-solving and coping strategies; psychological and emotional validation and support; and direction to community support and resources, as required. The Transition Counsellor utilised education, mindfulness practices, cognitive behavioural and narrative based therapeutic techniques as appropriate. Each one-hour session was tailored to the participant’s needs. To establish rapport and minimise withdrawal, the first three sessions were scheduled weekly if possible (weeks 1, 2, 3) and every three weeks thereafter (weeks 6, 9, 12). The final session (session 6, week 12) closed with a summary of the relevant insights and changes that the carer has made over the course of the sessions, and a review of any recommended solutions/techniques/external resources that have been discussed. This might include details of psychology or counselling services for ongoing support post-intervention or for the carer to discuss symptoms with their GP as appropriate. Ad-hoc sessions could also be provided where necessary. Training relating to the delivery of the RCTM (components, dose, resources) and the research study procedures (record-keeping, logs, data protection, confidentiality, reporting) was provided by the Trial Manager.

### Comparison group

Those in the comparison group received a check-in call from the Transition Counsellor following group allocation to see how they were, and if they would like any additional support e.g., details of Dementia Australia helpline. As per the US study protocol [23], a check-in call may partially adjust for the social engagement provided to the RCTM intervention group by the Transition Counsellor, whilst maintaining the integrity of the randomised control design [31].

### Both groups

Both groups received standard PDF help sheet information (freely available from Dementia Australia) including information for carers about planning for placement, coping with placement, developing caring partnerships with residential care staff, self-care for carers and managing feelings. The PDF information provided a control that did not replicate the active individualised counselling component provided in the intervention group. There is an ethical justification for providing standard information, freely available to dementia carers, to participants who may benefit as due to the resource constraints of the study we were unable to offer a wait-list control.

### Data collection

#### Baseline measures

Baseline data was collected following written consent to participate and before randomised group allocation. Data was collected using the Qualtrics online survey tool and participants were sent an email with a link to the survey for self-completion. Baseline data collected included: sociodemographic data relating to the carer and relative living with dementia; relationship to person living with dementia; length of time caring for their relative; living arrangements; how often they visit their relative if not co-residing; how involved they feel in making decisions about their relative; whether they have received any formal dementia education; whether they have received any counselling relating to their caring role; whether they have attended any support groups for carers; how they rate their own physical and mental health; length of time since ACAT approval; length of time on waitlist for residential care; dementia diagnosis of their relative.

### Process measures

The provision of intervention sessions was documented by the Transition Counsellor (date of session, time started and finished, topics covered, outcomes to follow-up for next session, any changes made and reasons for doing so) to determine “dose” of the intervention and to help inform subsequent sessions. 20% of audio-recorded intervention sessions were selected to check adherence to the protocol by the Trial Manager (as a measure of treatment fidelity). All participants were invited to take part in a 30-minute telephone/videoconferencing exit interview upon completion of the study to discuss their views (acceptability, feasibility, usefulness) on the RCTM intervention and/or PDF information package or check-in call. We also asked participants about suggestions for future improvements and wider implementation issues.

### Outcome measures

Outcome measures were collected using the Qualtrics online survey tool. As per baseline, participants were sent an email with a link to the survey for self-completion. Data was collected at baseline and 4 months post-baseline. Measures were limited to the following six instruments so as not to cause undue mental fatigue to participants:

- Perceived Stress Scale (PSS) [32];
- Caregiver Grief Scale (CGS) [33];
- Centre for Epidemiologic Studies Depression Scale (CES-D) [34];
- Caregiver Guilt Questionnaire (CGQ) [35];
- Geriatric Anxiety Inventory (GAI) [36];
- Support for Caring subscale of the Adult Carer Quality of Life Questionnaire (SFC) [37].
- Multiple outcomes are acceptable for use in feasibility studies, to help ascertain acceptability of measures for use in a future full scale RCT [38].

### Quantitative data analysis

Survey data was exported from Qualtrics into SPSS for analysis. At baseline, univariate analysis was carried out for each variable. The numerical summary for categorical variables was proportions (%) and for continuous variables, either the mean (standard deviation - SD) or median (inter-quartile range - IQR) measurement depending on tests of normality. Fisher’s Exact tests and two sample t-tests were conducted to determine if there were any statistical differences between the two groups at baseline. Outcomes were analysed using Repeated Measures ANOVA, with group (intervention versus control) as the between-subjects factor and time (baseline and 4 months post-baseline) as the within-subjects factor. *P* < 0.05 was used to indicate statistical significance.

### Qualitative data analysis

Interviews were audio-recorded and transcribed verbatim for qualitative analysis. Transcripts were imported into NVivo qualitative data analysis software and analysed using the ‘framework’ approach [39], informed by the MRC guidance for process evaluation of complex interventions [27]. The initial coding frame was developed as the first few interviews were completed, drawing both on the MRC domains of ‘contextual factors’, ‘mechanisms of impact’ and ‘implementation’ as well as emergent concepts from the participants themselves. Coding and analysis was conducted by authors DB and KW.

## Results

### Recruitment and retention

The ‘StepUp for Dementia Research’ registry released the study in phases to Australian States and Territories and matched with a total of 335 volunteers, most of whom did not reply to the first or second email approach. However, this was the most successful recruitment avenue, with 12/18 carers being recruited via StepUp. We were unable to recruit via ACAT teams as originally planned due to Australian State health organisation governance requirements to have site specific agreements in place for each recruiting site and with a principal investigator attached to each site. This was not a feasible approach for the study due to time and resource limitations.

**Fig. 2:**
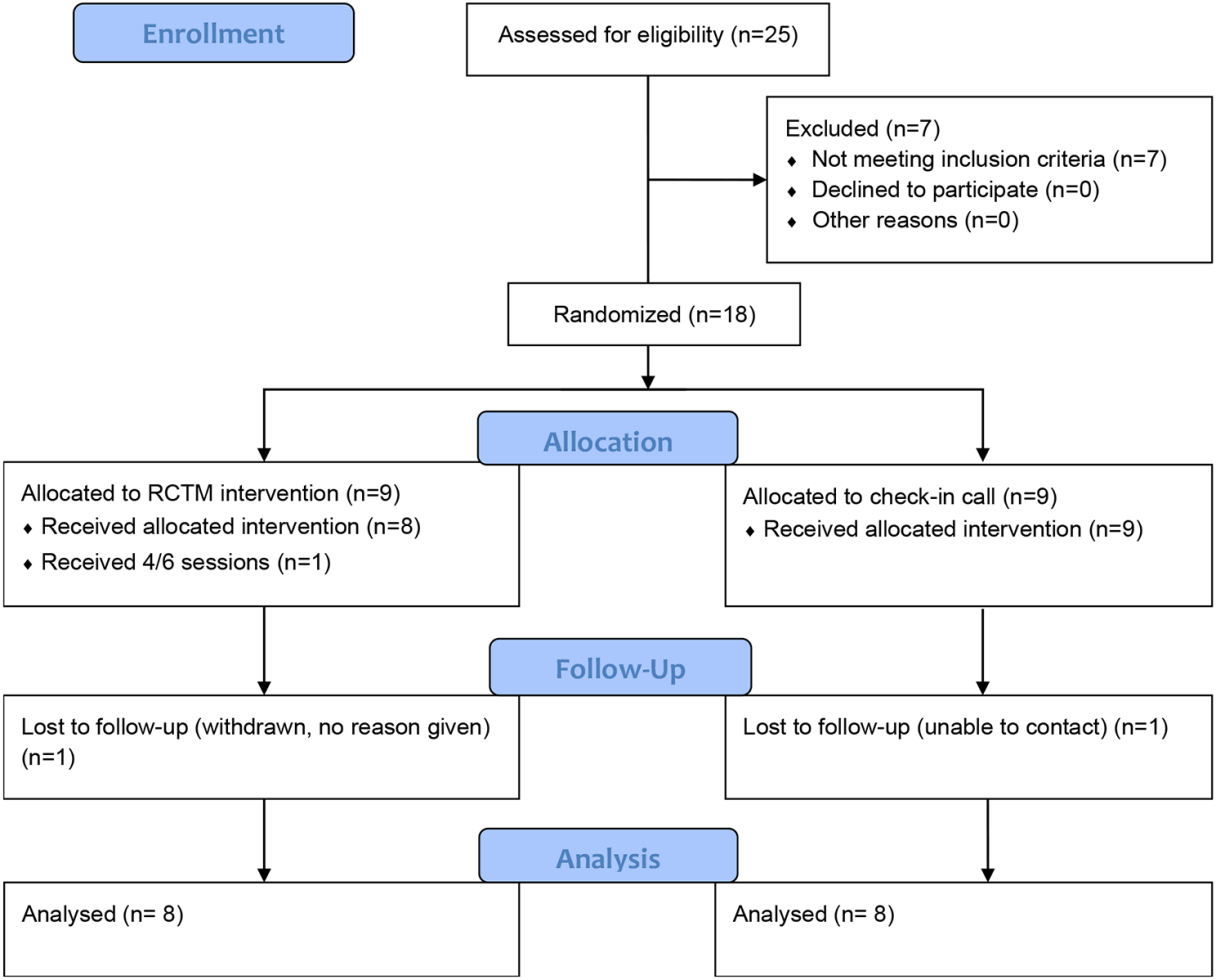
Consort diagram

Overall, we received expressions of interest from 29 family carers across five states (Queensland, New South Wales, Victoria, Western Australia, South Australia), however four of these did not respond to follow-ups following initial expression of interest. Of the 25 carers assessed for eligibility, seven carers were not eligible (one had not received ACAT approval and six had already moved their relative into permanent care). Whilst we aimed to recruit 30 family carers to the study, a total of 18 were recruited within the timeframe. Of these, one in the comparison arm was lost to follow-up and one in the intervention arm withdrew having completed 4/6 sessions (however, no reason for withdrawal was given as per HREC guidance). Participant flow through the study is shown in Fig. 2.

### Sample characteristics and baseline measures

Baseline data was collected for 18 participants, most of whom were female (94.4%). The mean age of carers was 60.5 years (SD = 10.45) and ranged from 31 to 77 years. Most participants were the child of the person living with dementia (*n* = 11) with other participants including spouse or partners (*n* = 5), daughter-in-law (*n* = 1) and granddaughter (*n* = 1). The majority (83.3%) of carers rated their own health as either excellent (*n* = 3), very good (*n* = 2) or good (*n* = 10). Nearly all participating family carers had received dementia education (94.4%), almost half (44.4%) had attended a support group and half (50%) had received counselling previously. There were eight participants (44.4%) who lived with the person with dementia and 10 (55.5%) who lived separately. Of those who did not live with the person living with dementia, over half visited their family member daily (*n* = 11), and the rest between daily and weekly. The median length of time since the person living with dementia had received ACAT approval for residential care, was nine months (ranging from 1 month to 5 years). For the seven people who were on a waitlist for residential care, the median time of being on the waitlist was 3 months (ranging from 1 to 14 months).

As well as demographic data, six outcome measures were collected for carers at baseline: PSS, CGS, CES-D, CGQ, GAI and SFC. For the PSS, almost three quarters (72%) of the sample scored between 14 and 26, which is considered to indicate moderate levels of stress. A further 22% scored over 26, indicating high levels of stress. For the CGS a score above 33 indicates feelings of grief in each subscale of Emotional Pain, Relational Loss, Absolute Loss, and a difficulty with Acceptance of Loss. Total scores indicate a high level of grief for 44% of participants who scored above 33 at baseline. The mean CES-D score showed 72% of participants scored 16 or above, indicating high levels of depressive symptoms at baseline. For the CGQ score, 83% of participants scored above 22 which is the clinical cut off score indicating a level of guilt that is likely to be associated with clinically significant symptoms such as depression, even though it is relatively low compared to the highest possible score. For the GAI a score of 9 and above indicates the presence of clinically significant anxiety. In this sample, 67% scored 9 or above. The mean SFC score indicated low to moderate perceived support (a higher score indicates greater perceived support).

There were no significant differences between the two groups on any of the variables at baseline, as shown in Table 1.

**Table 1:** Demographics and baseline scores on outcome measures by group

Variable	Control group (*n* = 9) mean (SD)	Intervention group (*n* = 9) mean (SD)	t	p (2-tailed)
Carer age (years)	64.00 (SD 7.18)	57.00 (SD 12.36)	1.469	0.161
Family member with dementia age	84.00 (SD6.36)	82.44 (SD 8.31)	0.446	0.662
Length of time caring (years)	5.27 (SD 2.83)	3.56 (SD 2.70)	1.317	0.206
Time on RACF waitlist (months)	4.75 (SD 6.24)	4.33 (2.08)	0.109	0.917
PSS (score from 0 to 40)	22.22 (SD 3.19)	23.11 (SD 7.13)	− 0.341	0.739
CGS (score from 11 to 55)	33.67 (SD 9.59)	33.44 (SD 9.49)	0.049	0.961
CES-D (score from 0 to 60)	24.00 (SD 7.92)	21.22 (SD 8.47)	0.719	0.483
CGQ (score from 0 to 88)	35.56 (SD 18.03)	42.00 (SD 12.92)	− 0.872	0.396
AC-QOL subscale (score from 0 to 15)	6.89 (SD 1.97)	6.22 (SD 2.91)	0.570	0.577
GAI (score from 0 to 20)	10.33 (SD 2.96)	8.89 (SD 5.35)	0.709	0.491

### Preliminary effects on outcomes

### Perceived stress (PSS)

**Fig. 3:**
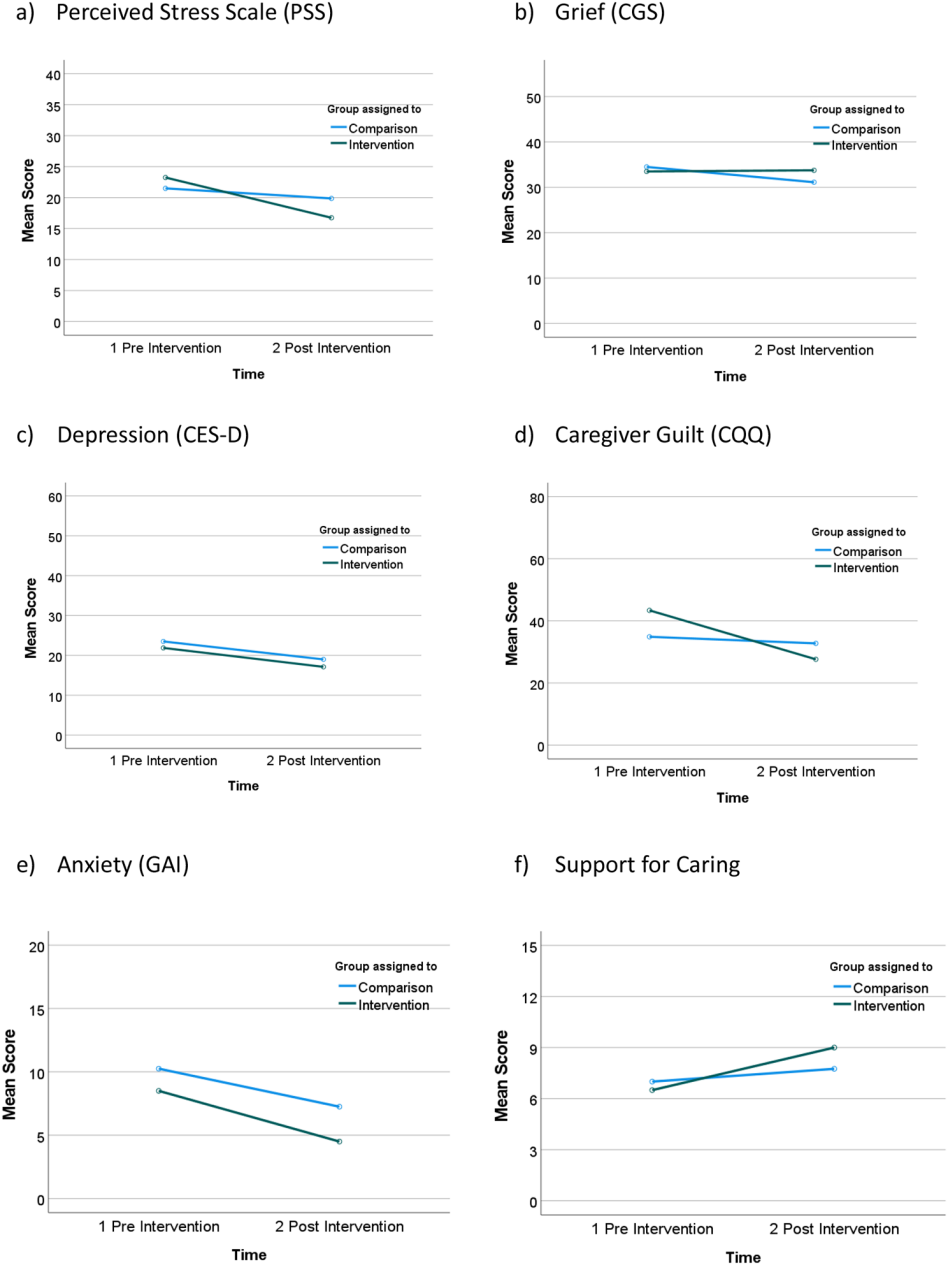
Scores over time (pre-intervention, post intervention) by group (intervention, comparison)

There were no significant differences between the two groups over time on total perceived stress scores. Due to the violation of the assumption of homogeneity of variance we were unable to use RM-ANOVA to analyse the interaction of time and group. However, there was a significant time effect with both groups improving over the study time period as indicated by a paired sample t-test, *t(14)* = 2.145, *p* = 0.05, *d* = 0.05. The mean score for the intervention group decreased from 23.25 to 16.75 and the comparison group decreased from 21.50 to 19.88 (See Fig. 3a).

#### Grief (CGS)

There were no statistically significant differences between the two groups over time on total grief scores, *F* (1,14) = 1.574, *p* = 0.230, partial *η*^*2*^ = 0.101. Grief scores remained high for both groups (See Fig. 3b).

#### Depression (CES-D)

There was a decrease in mean CES-D scores over time in both groups, however this was not quite statistically significant. *F (1,14)* = 4.062, *p* = 0.063, partial *η*^*2*^ = 0.225. The mean score in the intervention group decreased from 21.88 to 17.13 and in the comparison group from 23.50 to 19.00 (See Fig. 3c).

#### Guilt (CGQ)

There was a greater improvement in the guilt scores over time for the intervention group (mean score reducing from 43.37 to 27.63) compared to the comparison group (mean score reducing 34.88 to 32.75). However, this finding was not quite statistically significant, *F (1,14)* = 4.225, *p* = 0.059, partial *η*^*2*^ = 0.232, and guilt scores remained high in both groups. The overall decrease in guilt scores over time was significant, *F (1,14)* = 7.272, *p* = 0.017, partial *η*^*2*^ = 0.342 (See Fig. 3d).

#### Geriatric anxiety inventory (GAI)

There were no significant differences between the two groups over time on total anxiety scores. Due to the violation of the assumption of homogeneity of variance we were unable to use RM-ANOVA to analyse the interaction of time and group. However, there was a significant time effect with both groups improving over the study time period as indicated by a paired sample t-test, *t(14)* = 3.352, *p* = 0.005, *d* = 0.87. The mean score for the intervention group decreased from 8.50 to 4.50 and the comparison group decreased from 10.25 to 7.25 (See Fig. 3e).

#### Quality of life (ACQOL): support for caring

There was a statistically significant effect of time on mean Support for Caring scores, with both groups improving, *F (1,14)* = 9.031, *p* = 0.009, partial *η*^*2*^ = 0.392. The mean score for the intervention group improved from 6.50 to 9.00, while the mean score for the comparison group improved from 7.00 to 7.75 (See Fig. 3f).

### Feasibility of intervention delivery and adherence to protocol

With regard to the dose of the intervention, all but one participant received the six sessions as planned; one participant withdrew from the study after completing 4/6 of the planned sessions but did not give a reason for withdrawal. Of the participants that completed the program, the average total completion time was 12.88 weeks (range 11–15 weeks), with a longer time period required for two participants due to the Christmas holidays. The average session duration was 61 min (range 30–75 min). Regarding fidelity to the treatment manual, intervention adherence was high at 90% (20% audio-recorded sessions reviewed using a pre-defined audit form). No adverse events were recorded.

### Perceived usefulness and acceptability of the RCTM counselling, check-in call and PDF information

Exit interviews were held with eight participants from the intervention group and seven from the comparison group, following completion of the follow-up surveys. Interviews ranged from 20 to 78 minutes in length and were conducted either via telephone or via Zoom.

#### Contextual factors

During the study, one third of participants (three in the comparison group and three in the intervention group) placed their family member with dementia into permanent residential care. Timing of delivery of the RCTM program during the transition to permanent residential care was perceived to be feasible and acceptable, and deemed important. Some participants felt that counselling sessions provided earlier in the transition process or at the time of needing residential respite care were preferable.

*“I think if more people were aware of counselling before making the decision to put a loved one into permanent care, I think when their loved one maybe first goes into or just prior to going into respite, to give them an idea of what may lay ahead and– and help them not feel so either lost or guilty or both.” (P06)*.

Others thought that the counselling sessions would be beneficial following a hospitalisation or upon a realisation that they (as a family) could no longer cope with caring for their relative at home. The need for counselling to be offered at different stages of caring was raised by some participants, for example following diagnosis and following ACAT approval for residential care, or by stage of dementia.

#### Perceived benefits and mechanisms of impact

The RCTM counselling program was perceived to be beneficial by all the participants who received it. Participants preferred to speak to a trained counsellor and receive tailored information and advice rather than receive standard PDF information. Stated benefits included the focus of the counselling being specific to the long-term placement of their relative, emotional support and psychological change.

*“It was really, really helpful and, actually, through the programme it just helped me kind of, I mean, I already had broken down the steps, but it was more getting me, helping me to deal with the emotional side of it and just be able to move forward. I think it was holding me back a bit in progressing things. And, actually, during the counselling we ended up trialling some respite for mymum and it didn’t go very well the first time, so it was really great that I just happened to be in the middle of the counselling when this happened, so it was all leading up to when I had that support and then after it as well.” (P10)*.

One participant stated that they would have liked financial advice and geographically (e.g., Australian State) specific advice.

Mechanisms of impact included the skills of the Transition Counsellor, who was deemed to be knowledgeable about dementia and the move to residential aged care, empathetic and non-judgemental.

*“Well, I just think being in a space where they knew exactly what you were talking about. They were talking, this is an area that is their field. They know the feelings. They know all the things that we can talk through. There are ways to help you, you know, deal with things in the moment.” (P09)*.

Participants spoke about the benefit of having someone neutral to talk or ‘vent’ to, and the therapeutic rapport that was established. Emotional support and validation of feelings, such as allaying feelings of guilt, were deemed helpful if sometimes ‘confronting’.

*“I think it was very helpful. Especially in allaying guilt. I find guilt was the biggest thing about putting a loved one into care, because you think what could I have done better, or am I doing the right thing, and what if other people criticise me. There’s so much guilt that goes into it. But I think I’ve pretty much moved past the guilt stage now because I just look at him and I know that I couldn’t keep on going the way I was.” (P06)*.

Participants also valued the discussion of practical and emotional coping strategies, which were tailored to their specific needs each session.

*“Every time we talked she was able to give me strategies to address the things that I had some concerns about. She was also able to help me work out where I might have some concerns that I hadn’t quite figured out yet that I might have in the future.” (P05)*.

Some participants talked about how the RCTM program helped with their preparedness for placement and things to think about going forwards.

*“I think that some of the information that [counsellor] was able to give me has equipped me better now to be able to make some better decisions about what we might be looking at. She’s talked to me about what you need to be thinking about, what is it that you want to get from wherever you put the people into. What are the things that you must have? What are the things that would be nice to have? What are the things that you just certainly do not want to entertain? I think I probably hadn’t really thought about that until she talked through that with me one session…. That was, I think, probably one of the really good things that came out of that.” (P05)*.

#### Implementation

In terms of the implementation of the RCTM program, all participants found the delivery of the counselling sessions via videoconferencing (Zoom) to be acceptable. For some, use of videoconferencing was very familiar, and many deemed it more convenient and comfortable than in-person sessions. There were a few technical issues experienced, which could be ‘troubling’ but were easily resolved during the sessions. One participant expressed concern that videoconferencing might not be acceptable for older people who were not familiar with it and would need support to use it.

*“Yeah, it was good you could see the reaction and responses, so it was sort of a like a personal interaction. That was nice. Not just talking to a black screen or on the phone. I think that was really good. I know that not all people can do that but I’m able to so yeah, I think that was really good.” (P13)*.

The dose and spacing of sessions (6 sessions within 12 weeks), and with some flexibility (within the research parameters), was valued by participants to meet their individual needs. Some participants would have valued a follow-up call a few weeks or months after completion of the program.

*“It was actually really good for me. It worked out quite a good number and the spread was really good as well. So, it was enough ‘cause I can’t remember whether they were every two weeks, I think initially they were close together and then they spread out if I remember correctly. Yeah, so it was quite good, ‘cause those initial ones kind of got me going with the process and then I started to need a bit more time to complete some of the actions, so it worked out really well.” (P10).*

Conversely, few of the comparison group participants had comments about the check-in call received. Some participants could not remember receiving it or what was discussed; others felt that it was acceptable but had really wanted to receive the counselling.

Overall, the PDF help sheets that were emailed to participants in both groups, were considered ‘nothing new’, and often not read or remembered. Some preferred to access information through talking rather than reading. Others felt they were unable to keep up with the amount of information to read (from other sources or studies) or did not have time due to caring responsibilities. Most participants had received dementia education previously, either via the ‘understanding dementia’ massive open online course (MOOC) or from Dementia Australia.

*“It was stuff I already knew. It was at the level that I’d already was well aware of. There was nothing, or if there was anything new, probably my life is so busy, and my style of learning is more from talking rather than I’ve never been good looking at material and understanding it. I’m not a good learner in that way.” (P04)*.

## Discussion

We conducted a small feasibility study and pilot RCT of the RCTM intervention delivered via videoconferencing by a trained Transition Counsellor to family carers of a person living with dementia who had received ACAT approval and during the transition period to permanent residential care. The study aimed to assess feasibility of recruitment, retention and intervention delivery; acceptability of the intervention to family carers; and preliminary effects on carer perceived stress, grief, depression, guilt, anxiety, and support for caring.

Recruitment of sufficient numbers of family carers following ACAT approval was difficult within the time constraints of the study, resulting in a small sample of 18 participants. Whilst small sample sizes are often reported in pilot and feasibility studies, they decrease the statistical power of a study to detect an effect if there is one. Due to research governance restrictions, we were unable to recruit participants via ACAT teams, which would have been a more targeted recruitment approach. Whilst recruitment via the Australian StepUp for Dementia Research registry was successful, the resulting sample was mostly female, highly educated and had received a range of dementia education and carer supports prior to the study (e.g., via the dementia MOOC or Dementia Australia support organisation). However, we did recruit a mix of adult child and spousal carers with geographical representation from five Australian States. The lack of male carers is a limitation of this study, and discussions with stakeholders and our consumer advisory group indicates the need to potentially reframe the term ‘counselling’ for male carers [40], recruit via other avenues, and utilise male support workers.

Retention in the study was high at 89% which compares well with previous and similar studies [7, 18]. Delivery of the RCTM via videoconferencing was deemed feasible and acceptable with few technical issues, which reflects uptake and acceptance of videoconferencing telehealth more generally post-COVID [41, 42]. Treatment fidelity was high (90%), with flexibility to tailor the intervention to carers’ current problems or crises, as per the treatment protocol [23]. In terms of future implementation, this shows potential for the training of other dementia-specific counsellors in delivery of the RCTM and via videoconferencing, for example by current Dementia Australia counselling services.

At baseline, participants reported moderate to high levels of stress, grief, depressive symptoms, guilt, and anxiety, and low to moderate support for caring, reflecting the findings of other studies that the residential care transition period is a difficult, distressing and stressful time for family carers [12]. Preliminary effects of the RCTM at follow-up indicate that it may be beneficial in terms of alleviating feelings of stress and guilt and improving support for caring (with larger improvements gained over time than in the comparison group, albeit not statistically significant). This is important, as feelings of stress and guilt surrounding placement decisions are the most common emotional impacts reported by dementia family carers [4, 14]. It also echoes the findings from the qualitative component of the study, highlighting the importance of validation of feelings of guilt by the Transition Counsellor. Comparable reductions over time in depressive symptoms and anxiety were found in both the intervention and comparison group; significant time effects on depressive symptoms have been reported in other psychosocial intervention studies delivered following placement [18, 43]. It is important to note that during our study a third of participants (three in the comparison group and three in the intervention group) placed their family member with dementia into permanent residential care, which may have impacted findings. Feelings of grief, however, did not reduce in either group over time, and remained high at the end of the study. This finding differs from our previous study, where the RCTM was delivered to spousal carers post-placement and where the intervention group reported a greater ‘acceptance of loss’ than the comparison group at follow-up [18]. Variation in findings is likely a reflection of the different participant characteristics of the two study samples (i.e., mixed adult child and spousal carers versus only spousal carers), and/or the difference in timing of the intervention delivery (pre-placement versus post-placement).

From the qualitative component of the study, all participants in the intervention arm found the RCTM counselling program to be acceptable and particularly helpful in terms of the validation of their feelings of guilt and the discussions it facilitated regarding problem-solving and coping strategies related to the transition processes. The US RCTM study provides a detailed analysis of the types of carer guilt that can be experienced during the transition process, the differences between spousal and adult child experiences of guilt, and how the Transition Counsellor may tailor the program accordingly [14]. In the Australian study, the skills (empathy, non-judgmental) and dementia-specific knowledge of the Transition Counsellor were identified as important mechanisms of impact. Moreover, the therapeutic rapport that was established does not appear to have been affected by the delivery of the program via videoconferencing. However, a recent review of psychotherapy and counselling delivered via videoconferencing more generally, highlighted the need for more research in this area, particularly in relation to the ‘distance’ between therapist and client that is imposed by a screen, and the missed verbal and non-verbal responses and expressions this ensues [44].

Interestingly, neither the intervention nor the comparison group perceived the PDF informational help sheets to be beneficial (they were considered as ‘nothing new’), most likely as participants had previously been highly engaged with dementia education, information, and support. This is indicative of the participant selection bias introduced by the main recruitment pathway (i.e., those who sign up to the dementia research registry differ from other family carers) and as such is a limitation of the study. Such informational support may be beneficial for those carers who have not accessed such dementia education.

## Conclusions

This work adds to the emerging body of evidence regarding the acceptability and feasibility of delivering psychosocial interventions to family carers during the transition to permanent residential care of their relative living with dementia. It is the first study to apply the RCTM counselling intervention specifically within this pre-placement context and provides evidence of the acceptability and feasibility both of the timing of the intervention and its delivery via videoconferencing technology; an important finding with regard to improving access to dementia carer counselling interventions. Whilst targeted recruitment avenues (i.e., via aged care assessment teams and health services) were not feasible to achieve within this research study, rates of retention in the study were high and carers in the intervention group reported high levels of satisfaction with the counselling received. Preliminary effects on stress, guilt and support for caring are promising, however further research with a larger and more diverse sample of participants is needed.

## Data Availability

The datasets used and/or analysed during the current study are available from the corresponding author on reasonable request. Please contact Professor J. E. Gaugler for enquires regarding access to the RCTM intervention protocol.

## Abbreviations

ACAT: Aged Care Assessment Team
ANOVA: Analysis of Variance
CES-D: Centre for Epidemiologic Studies Depression Scale
CGS: Caregiver Grief Scale
CGQ: Caregiver Guilt Questionnaire
GAI: Geriatric Anxiety Inventory
HREC: Human Research Ethics Committee
MOOC: Massive Open Online Course
MRC: Medical Research Council
PSS: Perceived Stress Scale
RCTM: Residential Care Transition Module
RCT: Randomised Controlled Trial
SFC: Support for caring
TC: Transition Counsellor
